# Risk relationship between leukocyte telomere length and constipation: a Mendelian randomization study

**DOI:** 10.3389/fmed.2023.1177785

**Published:** 2023-09-13

**Authors:** Zhenfei Chi, Xuesong Bai, Zhe Zhang

**Affiliations:** ^1^Liaoning University of Traditional Chinese Medicine, Shenyang, Liaoning, China; ^2^Affiliated Hospital of Liaoning University of Traditional Chinese Medicine, Shenyang, Liaoning, China

**Keywords:** aging, leukocyte telomere length, constipation, Mendelian randomization study, GWAS, FinnGen

## Abstract

**Objective:**

Some epidemiological studies have investigated the associations between aging and constipation, yet their outcomes are inconclusive, so we strive to ascertain whether aging is the cause of constipation.

**Methods:**

We conducted a two-sample Mendelian randomization (MR) analysis using publicly accessible genome-wide association study (GWAS) summary statistics. As a marker of cellular and biological aging, we employed 15 single-nucleotide polymorphisms as instrumental variables for leukocyte telomere length (LTL) as exposure and a GWAS for constipation in the Finnish database as an outcome. To select the instrumental variables strongly associated with the phenotype, we eliminated confounding factors and direct effects outcomes to determine the causal relationship of exposure factors on the outcome; the analysis was mainly performed using the random-effect inverse variance weighting method, MR-Egger, weighted median, and sensitivity analysis of the results.

**Results:**

Random effect inverse variance weighted odds ratio = 1.035 (95% CI 0.907–1.180), but *p* = 0.612, which was not statistically significant. Other statistical methods, such as MR-Egger and weighted median, also yielded non-significant results.

**Conclusion:**

LTL as a proxy for aging does not necessarily indicate an increased likelihood of constipation. Further research is needed to explore the specific mechanisms of constipation.

## Introduction

Millions of people worldwide suffer from functional constipation (FC), a fast-growing public health problem in an aging society (1). A recent study estimated that 10.1% of the population has FC based on Rome IV (2). The prevalence of FC fluctuates across countries, ethnicities, and races; England has a prevalence of 8.6%, France 14.5%, Japan 16.6%, and China 22% in people over 60 years old. Constipation is associated with significant impairment of quality of life, is a burden to health care systems, and results in high individual healthcare costs. According to estimates from the UK National Health Service (NHS), the cost was £16.2 million or more from 2017 to 2018 (2). Studies have shown that FC might be associated with diet, medication, mood, and physical activity (3). Several global epidemiological surveys have been conducted: two surveys (4, 5) found the prevalence of FC increasing with age, while another three (6–8) showed the opposite, and two surveys (9, 10) found that FC did not differ by age. The causality of this relationship between aging and constipation is uncertain due to a lack of large samples from randomized controlled trials (RCTs) and strong evidence.

**Figure 1:**
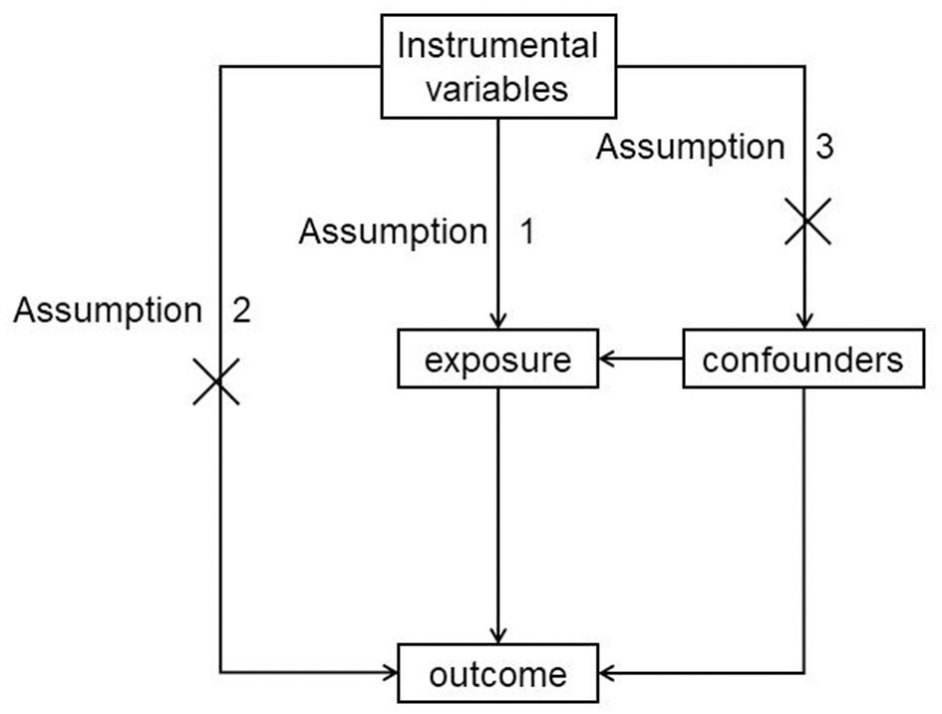
MR basic assumption.

Mendelian randomization (MR) is an epidemiological study design analogous to an RCT. Its purpose is to determine whether exposures can cause diseases using genetic variants as instrumental variables (IVs). There is no reverse causation bias because mutations are complete before birth and disease; there is no selection bias because MR also follows Mendel's second law, which implies that alleles of different genes assort independently of one another during gamete formation. Three major assumptions must be satisfied in MR: IVs should be strongly correlated with intermediate exposure, IVs should be independent of confounders, and IVs should have no directional horizontal pleiotropy effect on outcomes, as seen in Figure 1. MR is commonly considered a naturally occurring RCT, and it is a powerful study methodology to determine causality in the absence of RCTs (11).

Telomere length (TL) becomes shorter and shorter as cells divide because DNA polymerase cannot replicate the 3′ end of the DNA strand. Once the length of telomeres becomes critically short, the cell will enter a state of “replicative senescence.” After undergoing a limited amount of cell division, characteristic changes, including cell morphology, gene expression, and cellular functions, occur (12). TL is considered a biological marker of aging and is negatively correlated with age in some studies (13). It is easy to measure TL in peripheral blood leucocytes (LTL), so LTL always represents TL as an indicator of aging. In recent studies, LTL was widely relevant to age-related diseases and disorders such as coronary artery disease and certain cancers (14, 15). Between 44% and 86% of LTL levels can be attributed to genetics, while inter-individual LTL variation is largely determined before one's birth (16, 17). In previous studies, researchers proved the relationship between COVID-19 and skin aging using TL (18, 19).

Therefore, in this study, we aimed to establish a causal relationship between aging and FC using LTL as a proxy for aging. We conducted an MR analysis with FC as the “outcome” and LTL as the “exposure.”

## Methods

**Figure 2:**
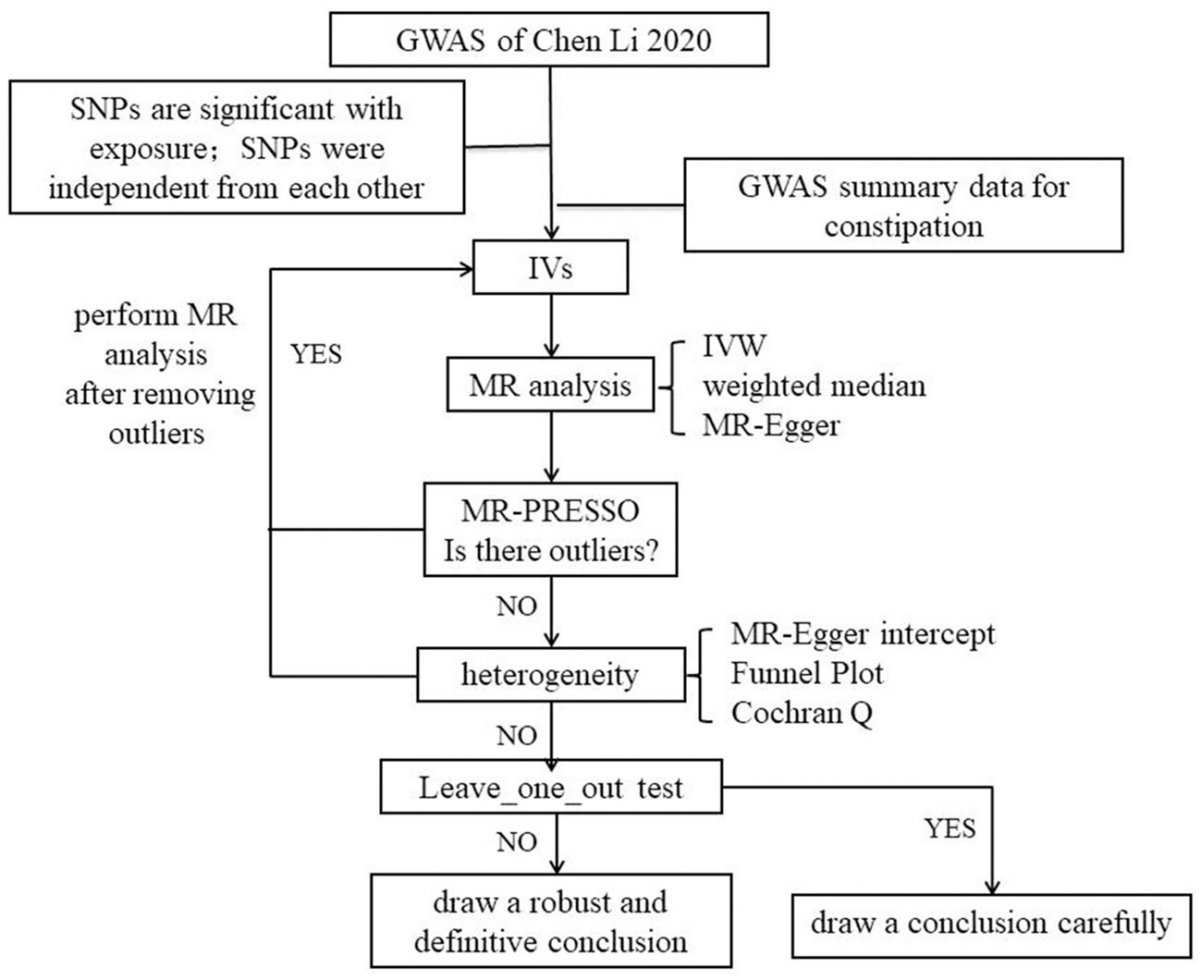
Flow chart.

An overview of the study design is drawn in Figure 2. We employed a two-sample MR study to explore potential causal relationships between exposure and outcome. We used data from a genome-wide association study (GWAS) to estimate the impact of the exposure (LTL) on the outcome (constipation). To identify IVs that are closely associated with LTL, we selected single-nucleotide polymorphisms (SNPs) from previously published GWAS databases and literature reports. The effects of these IVs on both exposure and outcome were obtained from two independent samples.

### Data sources

#### Exposure

Data were obtained from Chen Li's 2020 GWAS involving 78,592 individuals from the European Network for Genetic and Genomic Epidemiology (ENGAGE), the European Prospective Investigation into Cancer and Nutrition (EPIC), the Cardiovascular Disease (CVD) study, and the InterAct study (13). Outcome: Data were retrieved from FinnGen's GWAS with 26,919 cases and 282,235 controls (20). All the participants were of European descent.

#### Instrumental variables

A total of 20 SNPs at 17 genomic loci were reported in the GWAS by Chen Li 2020 (13); they were selected based on the following criteria: (1) SNPs are significant with exposure (*p* < 5e-08); (2) SNPs are independent of each other, we set the parameter *r*^2^ threshold to 0.001 and the kilobase pair (kb) to 10,000 to exclude interference in linkage disequilibrium; (3) SNPs are not related to outcome (*p* > 5e-08), so we removed the SNPs that were not matched in the GWAS of outcome; (4) we searched SNPs in PhenoScanner, and we removed the SNPs that had the potential to relate to constipation through other phenotypes; and (5) to avoid weak IV bias, F value was calculated according to a previous study (21). $F=\left(\frac{R^{2}}{{1-R}^{2}}\right)\left(\frac{n-k-1}{k}\right)$, *R*^2^ = 2 × (1−*MAF*) × *MAF*×β^2^; MAF = minor allele frequency, β = effect size, SE = standard error, n = sample size, and k = number of IVs.

#### Statistical method

We utilized the random-effects inverse-variance weighted (IVW) method as the main MR analysis (22), which integrates the Wald ratio estimates for each SNP on the outcome to obtain an aggregated causal estimate, resulting in the most significant statistical power. The random-effect IVW allows for the assumption that all instruments are ineffective if the overall horizontal pleiotropy remains balanced, and we also conducted complementary analyses based on MR-Egger regression (23) and weighted median (24, 25). In order to use the IVW method, it is assumed that all SNPs are valid instruments. The weighted median assumes that half of the genetic variants are valid IVs. MR-Egger regression assumes that none of the genetic variants are valid IVs (24). Based on the above assumptions, the efficacy of IVW is highest and that of MR-Egger is lowest in this article. All the abovementioned results are presented in the odds ratio (OR) and 95% confidence interval (95% CI).

#### Sensitivity analysis

We employed MR-Egger regression to evaluate the potential pleiotropic consequences of the SNPs as IVs. The MR-Egger intercept test could serve as a vital indication of whether the MR analysis results were driven by directional horizontal pleiotropy (26). In an MR-PRESSO analysis, the aim is to reduce heterogeneity in the causal effect estimate by eliminating SNPs that contribute excessively to heterogeneity. To achieve this, 1,000 iterations were set in the MR-PRESSO analysis. We utilized the IVW method and MR-Egger regression to identify any heterogeneity. The Cochran Q statistic was used to measure heterogeneity, and *p*-values below 0.05 indicated significant heterogeneity. Moreover, we performed a “leave-one-out” sensitivity analysis to detect possible influential SNPs by performing the MR while excluding each SNP in turn.

#### Ethics

The data utilized for this study were sourced from published GWAS datasets, thus forgoing the need for ethics committee approval. The included data sources, which were approved by the local ethics committee, conformed to the local regulations, and all participants provided informed consent.

The abovementioned statistical methods were performed on R (version 4.2.2 https://R-Forge.R-project.org/projects/) using the TwoSampleMR package (27).

## Results

Following the quality criteria and linkage disequilibrium filtering process (*p* < 5e-08, *r*^2^ < 0.001, kb = 10,000), we excluded four SNPs. One SNP that was missing when matched with the GWAS outcome was also excluded. Ultimately, a total of 15 SNPs were used as IVs. The *F*-value was calculated to be 200.56, which is significantly greater than the commonly accepted threshold of 10. This eliminates the possibility of weak instrument bias, and as such, we did not opt for proxy selection of unmatched SNPs. Refer to Table 1 for a list of the IVs used.

**Table 1:** Instrumental variables.

			**LTL**			**Constipation**		
**SNP**	**EA**	**OA**	**Beta**	**SE**	***p***	**Beta**	**SE**	***p***
rs10936600	T	A	−0.086	0.006	1.35E-46	−0.014	0.010	0.183
rs13137667	C	T	0.077	0.014	3.80E-08	0.012	0.030	0.691
rs228595	A	G	−0.029	0.005	6.63E-09	0.003	0.009	0.772
rs2302588	C	G	0.048	0.008	1.97E-09	−0.027	0.019	0.156
rs2736176	C	G	0.035	0.006	5.43E-09	0.013	0.010	0.214
rs3219104	C	A	0.042	0.006	2.56E-12	0.017	0.011	0.111
rs3785074	G	A	0.035	0.006	5.43E-09	−0.001	0.011	0.902
rs4691895	C	G	0.058	0.006	4.18E-22	0.002	0.012	0.876
rs59294613	A	C	−0.041	0.006	8.30E-12	0.014	0.011	0.194
rs62053580	G	A	−0.039	0.007	2.53E-08	−0.001	0.015	0.970
rs7194734	T	C	−0.037	0.006	6.97E-10	−0.025	0.012	0.043
rs75691080	T	C	−0.067	0.009	9.74E-14	−0.004	0.015	0.789
rs7705526	A	C	0.082	0.006	1.61E-42	−0.002	0.010	0.867
rs8105767	G	A	0.039	0.005	6.19E-15	0.003	0.010	0.793
rs9419958	C	T	−0.064	0.007	6.08E-20	0.034	0.014	0.020

### Causal effects of LTL on constipation

**Figure 3:**
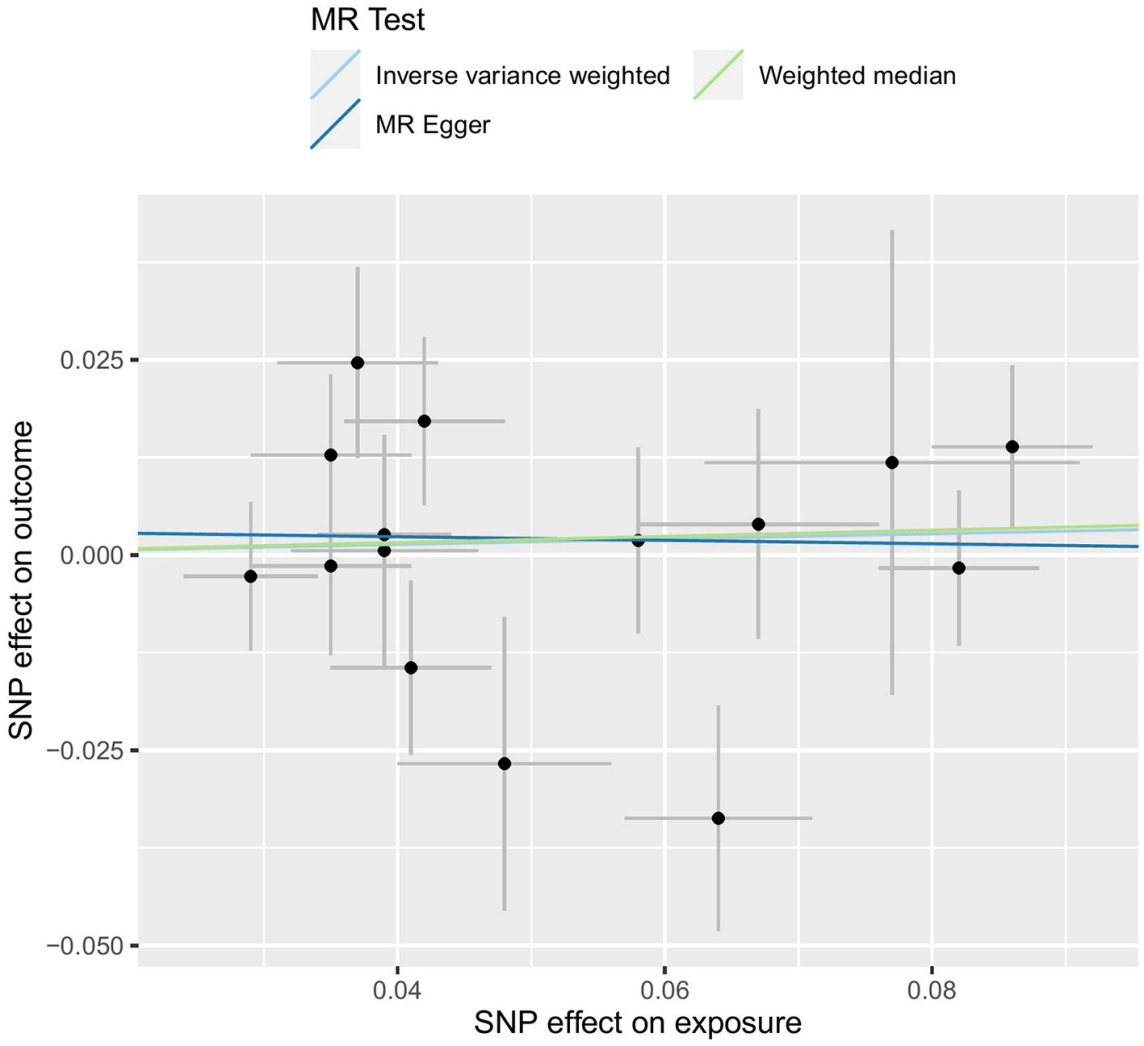
MR test.

Our analysis, which used the IVW approach, did not demonstrate any significant relationship between LTL and an increased risk of constipation (OR = 1.035, 95% CI 0.907–1.180; *p* = 0.612). The weighted median OR was 1.040 (95% CI 0.893–1.213), with a *p*-value of 0.612, while the MR-Egger OR was 0.978 (95% CI 0.668–1.431), with a *p*-value of 0.910. Additionally, there was no statistically significant or suggestive correlation found between genetic susceptibility to LTL and constipation (all *p* > 0.05). Therefore, our conclusion is that LTL is unlikely to be a causal factor in constipation. The results are shown in Table 2 and Figure 3.

**Table 2:** MR results.

**Method**	**nSNP**	**OR**	**SE**	***p*-value**	**95% CI**
Inverse-variance weighted	15	1.035	0.067	0.612	0.907–1.180
Weighted median	15	1.040	0.078	0.612	0.893–1.213
MR-Egger	15	0.978	0.194	0.910	0.668–1.431

### Sensitivity analysis

**Figure 4:**
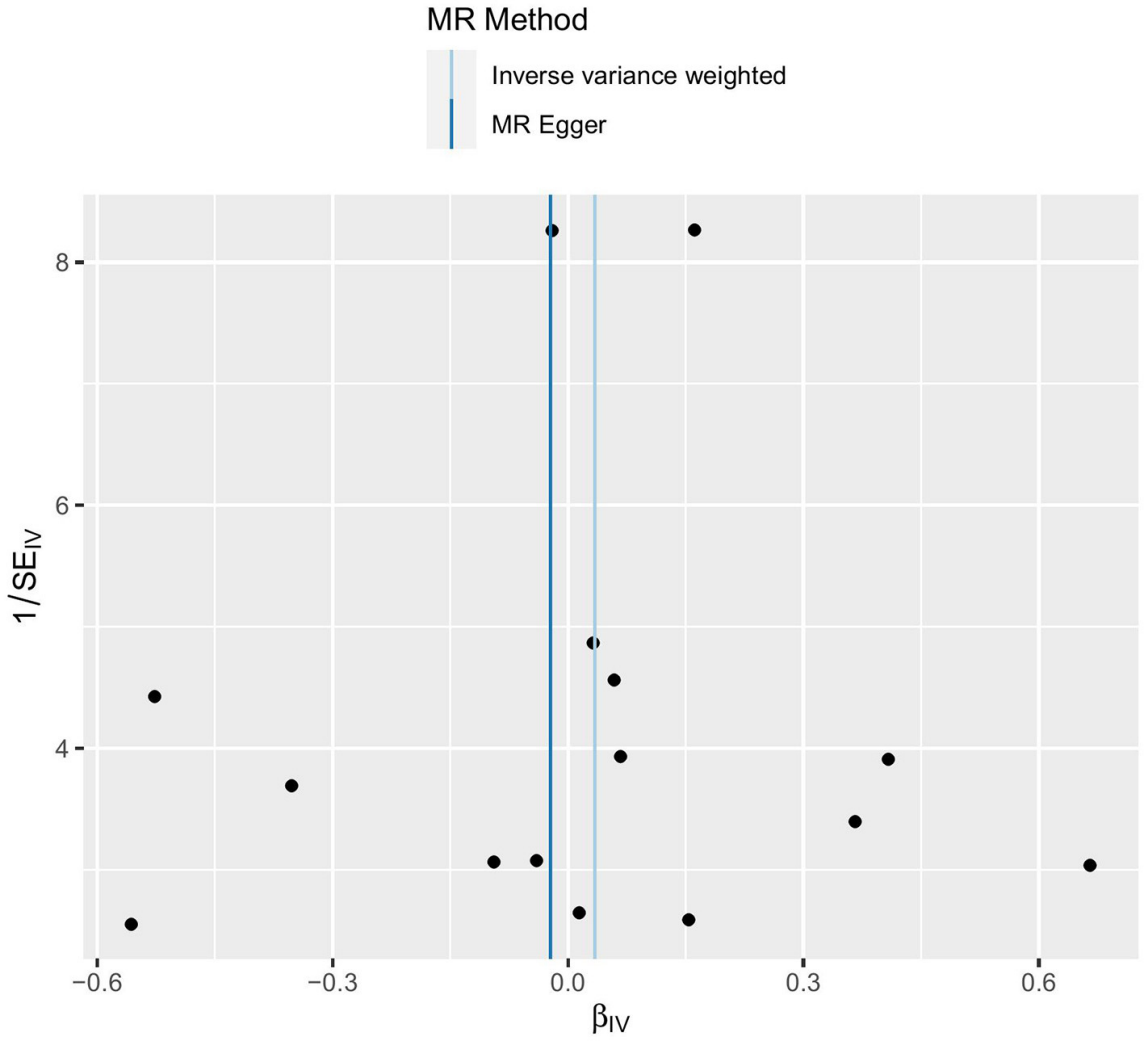
Funnel plot.

**Figure 5:**
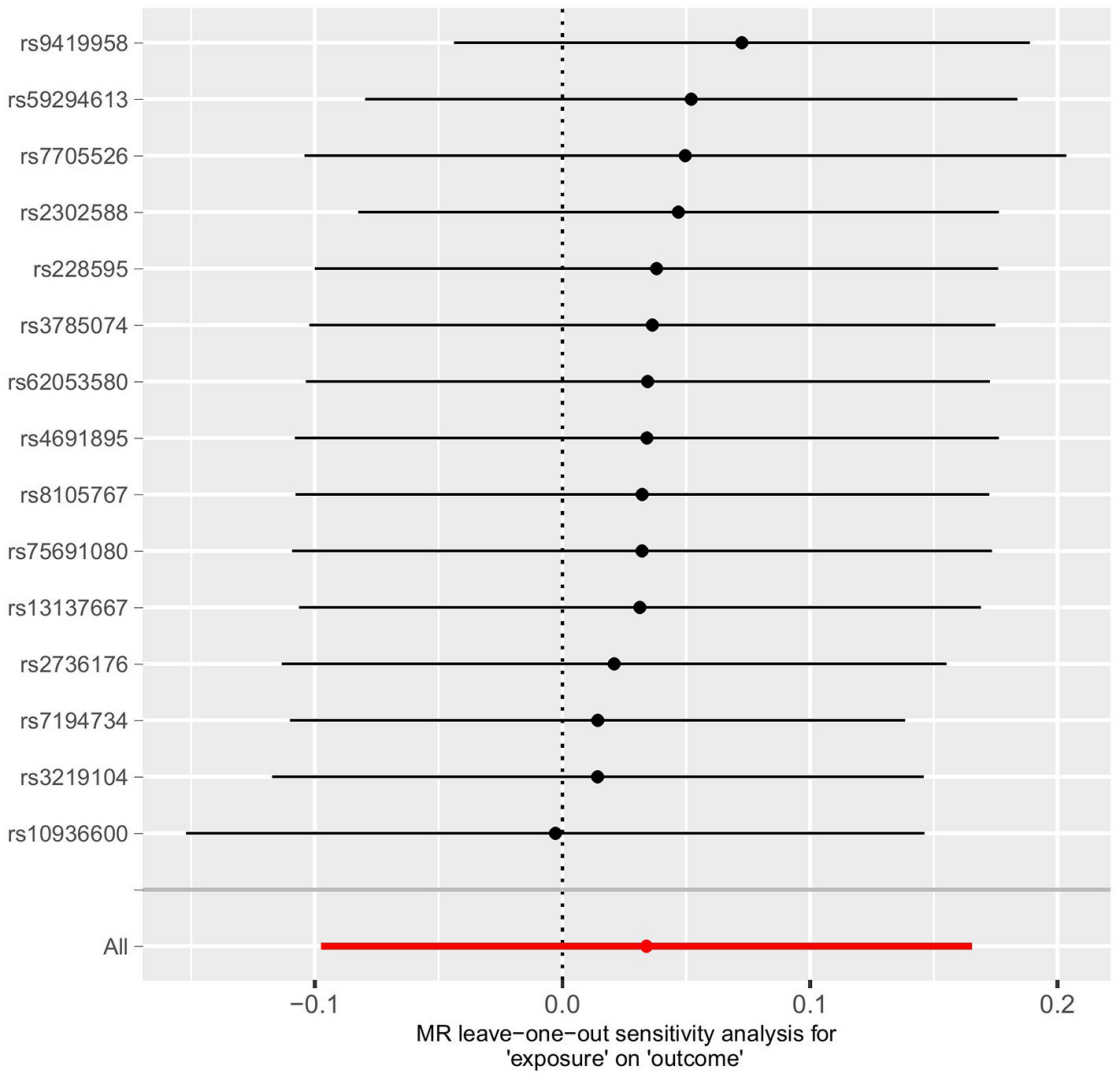
Leave-one-out test.

To assess the robustness of the results, several sensitivity analyses were conducted, including the MR-Egger intercept test, Cochran Q test, MR-PRESSO test, funnel plot (Figure 4), and Leave-one-out test (Figure 5). The MR-Egger intercept test yielded a *p*-value of 0.761, the Cochran Q test yielded a *p*-value of 0.158, and the MR-PRESSO test yielded a *p*-value of 0.620. All *p*-values were >0.05. The MR-Egger intercept test indicated the absence of horizontal pleiotropy in the analysis, thereby ruling out any interference, influence, or mediation effects of variables at different levels or possible MR effects. The Cochran Q test and MR-PRESSO test confirmed the absence of SNP-induced heterogeneity. Furthermore, in the Leave-one-out test (Figure 5), the risk estimate did not change significantly after each removal of a single SNP, demonstrating that no specific SNP was essential to causal association. Additionally, as shown in the funnel plot (Figure 4), the effect size variation around the point estimate was symmetric, indicating no significant horizontal pleiotropy.

## Discussion

Our study goal was to determine the causal link between FC and aging using LTL as a proxy for aging. Through the application of MR analysis, FC was considered the “outcome,” while LTL was treated as the “exposure” (28). The result of the random-effects IVW method, which assumes all SNPs are valid instruments and has the highest efficacy, was OR = 1.035 (95% CI 0.907–1.180), but *p* = 0.612, which was not statistically significant. The result of the weighted median, MR-Egger, was congruous with IVW. All MR analyses demonstrated that LTL does not cause constipation.

In a previous study, Nicholas J. Talley conducted a cross-sectional study in Olmsted County in 1991 (4) and concluded that people aged 60–64 years may have contributed to a higher FC risk than those aged 50–59 years. Douglas A. Drossman also conducted a cross-sectional study all over the United States (7), but the conclusion was the opposite, with the author explaining that sociocultural and behavioral factors might influence the tendency to amplify or generalize these sensations. There was an epidemiological investigation by P. Pare in 2001 in which he conducted a random digit dial national survey through a computer-assisted telephone interviewing (CATI) system, and concluded that FC affects the young and the elderly with similar frequency. The author explained that different sampling methods, definitions of FC, or dietary and cultural characteristics of the study populations might have produced this result. All of the above are cross-sectional studies, and the results did not determine whether aging causes constipation. The relationship between aging and constipation is unclear due to bias and confounders such as medication use, depression, dietary habits, study design, and small sample sizes. Thus, we need stronger levels of evidence, such as RCTs or MR.

Aging is a natural process that often involves the loss of telomeres. This can result in various problems, such as weakened immune systems, diminishing immune cells, and increased susceptibility to age-related neurological diseases due to inflammation and autoimmunity (29). Telomeres are crucial for protecting cells during activities such as cell division, degradation, and repair. They are comprised of guanine-rich sequences at the end of chromosomes, making them highly susceptible to oxidative stress and inflammation (30). TL is closely linked to aging and is influenced by environmental, genetic, and behavioral factors (31). Chronic inflammation and oxidative stress can accelerate telomere depletion. Previous research has found a connection between TL decline and an increased presence of DNA damage and protein oxidation markers (32). However, while chronic inflammation and oxidative stress have been linked to constipation (33, 34), there is insufficient evidence to support a connection between TL and constipation.

The IVs used in this study are based on SNPs reported in previous research to have a significant impact on LTL. However, one SNP was missing during the matching process; hence, we calculated the F-statistic to avoid bias from weak instruments. The effects of these IVs on both exposure and outcome were obtained from two independent samples. We tried to minimize heterogeneity and exclude confounding factors before using the data, but individual differences still cannot completely eliminate potential influencing factors. In the presence of potential heterogeneity, random effects models can allocate weights in a more balanced way than fixed effects models and are better able to explain the potential heterogeneity of genetic variations or adjust for unmeasured confounders (35). Moreover, it is well-known that the efficiency of MR-Egger regression is much lower than IVW (36), so we ultimately chose the IVW method under the random effects model as the main analysis method. The result was OR = 1.035, 95% CI 0.907−1.180, which did not support the hypothesis that LTL causes constipation. In sensitivity analysis, the MR-Egger intercept test indicated the absence of horizontal pleiotropy in the analysis, and the Cochran Q test and MR-PRESSO test confirmed the absence of SNP-induced heterogeneity. Finally, we concluded that LTL is not the cause of constipation.

It is imperative to acknowledge that constipation in the elderly is influenced by a myriad of factors. While LTL may be associated with certain facets such as environmental air quality (37), organ functionalities (38), weight fluctuations (39), and major depressive disorder (40), it is vital to note that the mere utilization of LTL may not significantly impact the overall incidence of constipation. Thus, it would be premature to surmise that the process of senescence itself augments the likelihood of constipation. Observational studies have yielded incongruous outcomes, which can be attributed to sundry rationales. The augmented risk of constipation in the elderly, as reported in certain investigations, could be ascribed to factors such as exercise proclivities, medication utilization, insufficient aqueous intake, malnutrition, acute and chronic ailments, stroke-related complications (41), alterations in the composition of gut microbiota (42), psychological factors (43), and other contributory variables. These biological pathways, rather than the innate aging process itself, plausibly play a role in the development of constipation within the elderly populace. To attenuate bias and attain a comprehensive comprehension of the factors influencing constipation, prospective studies should explore additional latent factors beyond LTL. This will ensure a more precise assessment of the relationship between constipation and the various contributory factors within the geriatric population.

## Advantages and limitations

### Advantages

The merit of utilizing the MR analysis for this study is its ability to draw causal inferences regarding the correlation between TL and constipation while mitigating the influence of confounding factors and reverse causality. By means of genetic variants as IVs, reliable estimations regarding the effect of TL on constipation were obtained. This study's ingenuity lies in its emphasis on the negative outcome of the MR analysis. Instead of corroborating an established relationship between TL and constipation, we discovered no indication of a causal link between these two variables. This underscores the significance of deploying rigorous research methods to scrutinize potential causal associations, and our study contributes valuable insights into the possible mechanisms underlying the onset of constipation. Furthermore, it illustrates the usefulness of the MR analysis in disentangling causality in intricate relationships. The database boasts a vast sample size; both the exposure and outcomes in this study were European, precluding racial bias.

### Limitations

One of the limitations of this study is that constipation was treated as a binary variable in GWAS, and the diagnosis and enrollment were performed by the Finn database, which did not consider the severity of constipation. Additionally, GWAS is mostly carried out in Europe, so the extrapolation of our findings to other populations is uncertain. Furthermore, the physiological mechanisms underlying the relationship between TL and constipation are complex and cannot be fully captured by the MR model. Another limitation is that the IVs used in this study were based on SNPs that have been reported in previous studies to have a significant impact on LTL, but they had missing values during the matching process. Even though we calculated the F-statistic to avoid bias from weak instruments, the possibility of bias cannot be completely ruled out. Finally, the study could not be stratified according to the degree of disease or sex due to the use of aggregated data, although previous studies have shown that constipation is more prevalent in women than men (44). Further studies are needed to determine the underlying mechanism of constipation.

## Conclusion

LTL as a proxy for aging does not necessarily indicate an increased likelihood of constipation. Further research is needed to explore the specific mechanisms of constipation.

## Data availability statement

Publicly available datasets were analyzed in this study. This data can be found here: FinnGen's GWAS (https://r7.finngen.fi/).

## Author contributions

ZC is responsible for writing the paper and collecting related data. XB is in charge of the statistical segment of the paper. ZZ is responsible for reviewing and providing statistical guidance. All authors contributed to the article and approved the submitted version.
